# The Role of Series Cholecystectomy in High Risk Acute Cholecystitis Patients Who Underwent Gallbladder Drainage

**DOI:** 10.3389/fsurg.2021.630916

**Published:** 2021-02-15

**Authors:** Chi-Chih Wang, Ming-Hseng Tseng, Sheng-Wen Wu, Tzu-Wei Yang, Wen-Wei Sung, Yao-Tung Wang, Hsiang-Lin Lee, Bei-Hao Shiu, Chun-Che Lin, Ming-Chang Tsai

**Affiliations:** ^1^Institute of Medicine, Chung Shan Medical University, Taichung, Taiwan; ^2^School of Medicine, Chung Shan Medical University, Taichung, Taiwan; ^3^Division of Gastroenterology and Hepatology, Chung Shan Medical University Hospital, Taichung, Taiwan; ^4^Department of Medical Informatics, Chung Shan Medical University, Taichung, Taiwan; ^5^Division of Nephrology, Chung Shan Medical University Hospital, Taichung, Taiwan; ^6^Department of Urology, Chung Shan Medical University Hospital, Taichung, Taiwan; ^7^Division of Pulmonary Medicine, Chung Shan Medical University Hospital, Taichung, Taiwan; ^8^Department of Surgery, Chung Shan Medical University Hospital, Taichung, Taiwan; ^9^Department of Internal Medicine, China Medical University Hospital, Taichung, Taiwan; ^10^School of Medicine, China Medical University, Taichung, Taiwan

**Keywords:** gallbladder drainage, cholecystectomy, acute cholecystitis, recurrent biliary event, medical expenses

## Abstract

**Background:** Cholecystectomy (CCY) is the only definitive therapy for acute cholecystitis. We conducted this study to evaluate which patients may not benefit from further CCY after percutaneous transhepatic gallbladder drainage (PTGBD) has been performed in acute cholecystitis patients.

**Methods:** Acute cholecystitis patients with PTGBD treatment were selected from one million random samples from the National Health Insurance Research Database obtained between January 2004 and December 2010. Recurrent biliary events (RBEs), RBE-related medical costs, RBE-related mortality rate and an RBE-free survival curve were compared in patients who accepted CCY within 2 months and patients without CCY within 2 months after the index admission.

**Results:** Three hundred and sixty-five acute cholecystitis patients underwent PTGBD at the index admission. A total of 190 patients underwent further CCY within 2 months after the index admission. The other 175 patients did not accept further CCY within 2 months after the index admission. RBE-free survival was significantly better in the CCY within 2 months group (60 vs. 42%, *p* < 0.001). The RBE-free survival of the CCY within 2 months group was similar to that of the no CCY within 2 months group in patients ≥ 80 years old and patients with a Charlson Comorbidity Index (CCI) score ≥ 9.

**Conclusions:** We confirmed CCY after PTGBD reduced RBEs, RBE-related medical expenses, and the RBE-related mortality rate in patients with acute cholecystitis. In patients who accepted PTGBD, the RBE and survival benefits of subsequent CCY within 2 months became insignificant in patients ≥ 80 years old or with a CCI score ≥ 9.

## Introduction

Cholelithiasis is one of the most popular diseases with increasing prevalence and substantial burden on healthcare resources (1, 2). Because the abundant access to food worldwide increases the risk of obesity, the incidence rates of cholelithiasis grow accordingly (3, 4). Cholecystitis refers to inflammation of the gallbladder, and it can be defined as acute or chronic cholecystitis by the duration of the disease. Acute acalculous cholecystitis accounts for only <10% (5, 6) of all cholecystitis patients. Acute cholecystitis is a complication of gallstone disease and typically develops in patients with a history of symptomatic gallstones (7, 8).

After the diagnosis of acute cholecystitis, evaluations of the disease severity are necessary (9) to guide clinical management, such as early cholecystectomy (CCY) or percutaneous transhepatic gallbladder drainage (PTGBD), especially in critically ill patients (10, 11). According to the timing of CCY, CCY can be further classified as early or delayed CCY, and early CCY is a better choice than delayed CCY in terms of hospital stay and the medical expenses of treatment (12). Compared to delayed CCY, early CCY for cases within 72 h of symptom onset was associated with lower mortality rates and complication rates in the majority of cases in previous literature (12–14). Some references show that delayed CCY has a higher proportion of laparoscopy (15) and a lower complication rate (16). Although CCY is the only definitive therapy for acute cholecystitis (9, 17, 18), there are patients who cannot tolerate or do not want surgical intervention despite the benefits of early or delayed CCY. Although the role of PTGBD in acute cholecystitis remains a debate (19, 20) compared to conservative treatment, PTGBD is useful for managing high surgical risk patients and patients with severe comorbidities (9, 21, 22). Currently, there is no strong evidence to suggest surgery timing after patients with acute cholecystitis have undergone PTGBD (23–25), but CCY could decrease the likelihood of recurrent biliary events (RBEs) (26) after successful PTGBD intervention in these patients. As a result, patients with acute cholecystitis, who were successfully treated by PTGBD, should accept further early, interval, or delayed CCY to prevent future RBEs.

As our recognition, acute cholecystitis patients who have high comorbidity or were extremely old may not benefit from further CCY. We used the Charlson Comorbidity Index (CCI) (27, 28) to represent the level of comorbidities in patients in our study, which obtained from the Taiwan National Health Insurance Research Database (NHIRD). There are no current guidelines for the optimal timing for performing CCY after PTGBD, and only few real-world studies suggest the timing of CCY (29) due to surgical complications. We attempted to evaluate the benefits of CCY after patients with acute cholecystitis underwent PTGBD and whether the benefits disappear due to severe comorbidities or old age in a retrospective database cohort study.

## Methods

Our study was approved by the Institutional Review Board (IRB) of Chung Shan Medical University Hospital in Taiwan. All methods were performed in accordance with the relevant regulations and under the surveillance of the IRB of Chung Shan Medical University Hospital.

### Study Design

This study was a population-based retrospective cohort study based on Taiwan's NHIRD, which covers more than 99% of the entire population (30). The NHIRD has been described in detail in previous studies (31–33).

**Figure 1:**
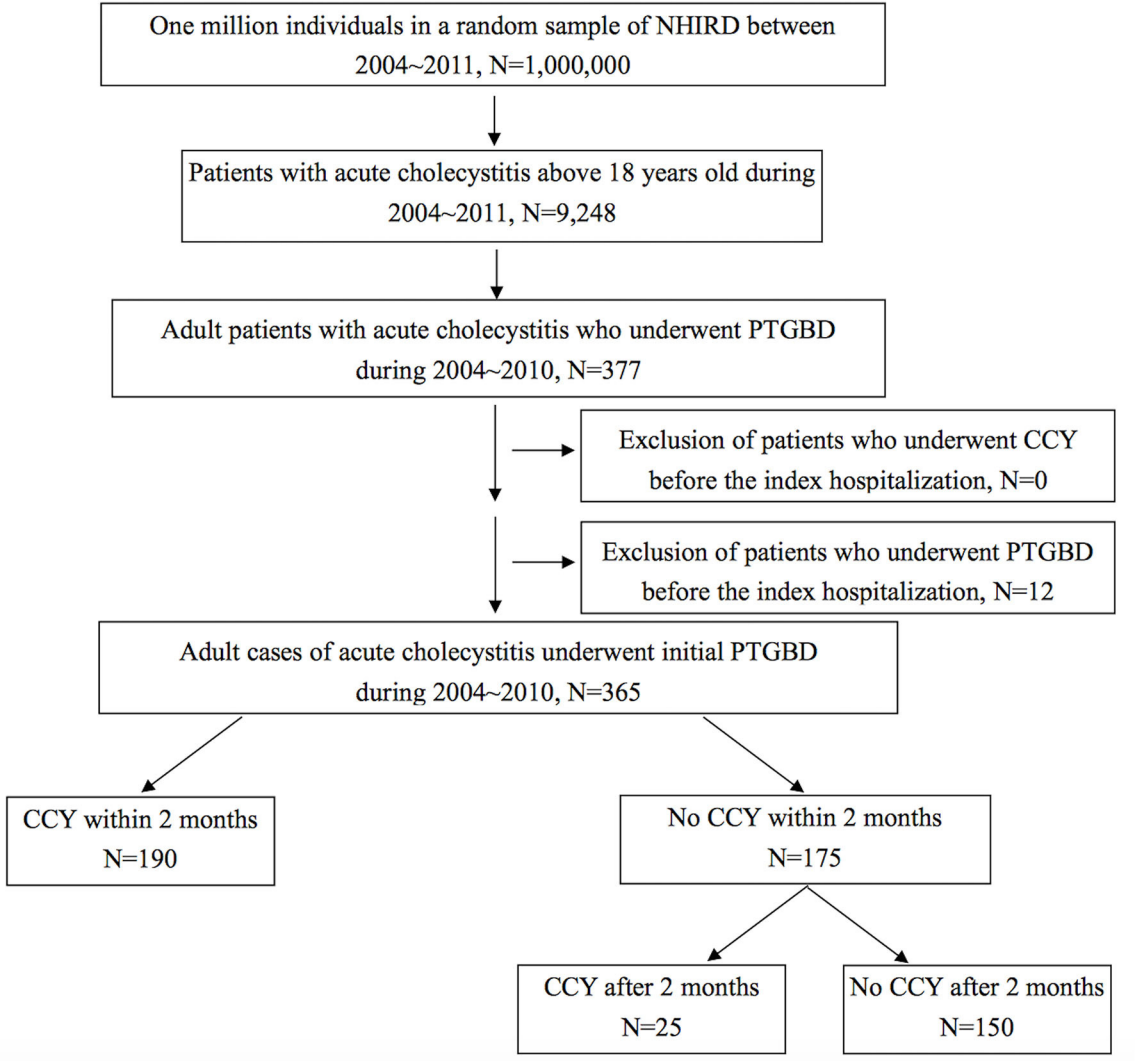
Flow chart for case selection from a database of one million nationwide representatives in Taiwan. NHIRD, National Health Insurance Research Database; PTGBD, percutaneous transhepatic gallbladder drainage; CCY, cholecystectomy.

Cholelithiasis cases were selected from one million random samples from the NHIRD between 2004 and 2011 using the codes of International Statistical Classification of Diseases and Related Health Problems, 9th Edition (ICD-9) recorded by emergency room (ER) visits or admissions. Patients with acute cholecystitis were selected using ICD-9 codes documented in 2004–2010 to ensure the follow-up period is more than 1 year, and then, we identified patients who underwent PTGBD (procedure code 33106B) during an index admission or during an ER course 3 days before admission. Patients who had previously undergone CCY or PTGBD between the index admission and 2004 were excluded. The observation period was selected from the index admission to December 2011 or the expired date of patients. A total of 365 patients with acute cholecystitis who underwent PTGBD were selected, and 190 of these patients accepted CCY within 2 months (60 days) after the index admission. The other 175 patients with acute cholecystitis who underwent PTGBD during the index admission did not accept CCY 2 months after the index admission. Twenty-five patients in the no CCY within 2 months group eventually underwent CCY during the follow-up period. We compared age, gender, baseline CCI score, the proportion of laparoscopic and open CCY procedures, the total follow-up time, time to events, RBEs, overall mortality, RBE-related mortality, and the total medical expenses for RBEs between these two groups. RBE-related mortality was defined as mortality events due to RBEs during hospitalization or within 5 days after discharge. The design of this study is shown in Figure 1.

An economic analysis of the costs of the index admission, CCY events, ER visits and hospitalization due to RBEs, and the total medical expenses were compared between the CCY within 2 months group and the no CCY within 2 months group for patients with acute cholecystitis who initially underwent PTGBD under Taiwan's national health insurance system.

### Data Processing and Statistical Analysis

The one million individuals who represent the nationwide population between 2004 and 2011 in Taiwan were processed using Microsoft SQL Server 2008 R2 (Microsoft Corporation, Redmond, WA, USA) with the SQL programming language. Statistical analysis was performed using OpenEpi: Open Source Epidemiologic Statistics for Public Health, version 3.01 (40). Kaplan–Meier survival analyses were conducted using SPSS version 19.

The data obtained from the study were processed using Chi-Square (χ^2^) tests for categorical variables, one-way analyses of variance (ANOVA) for continuous variables, and log-rank (Mantel–Cox) tests for disease-free survival curves. A two-tailed *P*-value of 0.05 was considered statistically significant.

## Results

A total 9,248 adult patients with acute cholecystitis were selected from one million samples from January 2004 to December 2011. Because the follow-up period should be at least 1 year, we included 377 acute cholecystitis patients who underwent PTGBD between January 2004 and December 2010. Twelve patients were excluded because of previous PTGBD before the index admission. The data for 365 acute cholecystitis patients who underwent PTGBD at the index admission were collected. A total of 190 patients with a mean age of 68.42 ± 13.17 years underwent further CCY within 2 months after the index admission. The other 175 patients, with a mean age of 72.67 ± 13.75 years, did not accept further CCY within 2 months after the index admission. The baseline CCI score, 5.77 ± 3.41 in the CCY within 2 months group and 7.79 ± 4.10 in the no CCY within 2 months group, was significantly higher in the no CCY group (*p* = 0.01). As for the components of the CCI, the proportions of patients with congestive heart failure (10.53 vs. 25.14%, *p* < 0.01), hemiplegia or paraplegia (0 vs. 3.43%, *p* = 0.04), and chronic kidney disease (9.47 vs. 25.14%, *p* < 0.01) were significantly higher in the no CCY group. The total follow-up duration was significantly longer in the CCY within 2 months group than in the no CCY within 2 months group (39.68 ± 23.47 vs. 23.47 ± 20.26, *p* = 0.047). The detailed baseline demographic data are shown in Table 1.

**Table 1:** Demographic data of patients underwent percutaneous transhepatic gallbladder drainage.

**Study group**	**CCY within 2 months** ***N*** **=** **190**		**No CCY within 2 months** ***N*** **=** **175**		***P*-value**
	***N*; Mean**	***SD*; %**	***N*; Mean**	***SD*; %**	
**Age (*****SD*****)**	68.42	13.17	72.67	13.75	0.57
**Gender**					0.20
Male	117	61.58	96	54.86	
Female	73	38.42	79	45.14	
**Follow up time (months)**	39.68	23.47	23.47	20.26	0.047
**Comorbidity**					
**CCI score (*****SD*****)**	5.77	3.41	7.79	4.10	0.01
MI	7	3.68	13	7.43	0.29
CHF	20	10.53	44	25.14	<0.01
Peripheral vascular disease	12	6.32	18	10.29	0.38
CVA	38	20.00	55	31.43	0.43
Dementia	22	11.58	28	16.00	0.47
CPD	64	33.68	70	40.00	0.45
Rheumatologic disease	6	3.16	4	2.29	0.88
PUD	101	53.16	90	51.43	0.95
Mild liver disease	40	21.05	41	23.43	0.86
DM	71	37.37	85	48.57	0.10
DM with complication	19	10.00	27	15.43	0.29
Hemiplegia or paraplegia	0	0.00	6	3.43	0.04
CKD	18	9.47	44	25.14	<0.01
Malignancy (including leukemia and lymphoma)	31	16.32	38	21.71	0.42
Liver cirrhosis	6	3.16	13	7.43	0.18
Metastatic solid tumor	10	5.26	19	10.86	0.14
HIV	0	–	0	–	N.A.

*SD, standard deviation; CCI score, Charlson Comorbidity Index score; MI, Myocardial infarction; CHF, congestive heart failure; CVA, Cerebrovascular accident; PUD, Peptic ulcer disease; CPD, Chronic pulmonary disease; DM, Diabetes; CKD, chronic kidney disease; HIV, human immunodeficiency virus; N.A., not applicable*.

Reflecting the culture in Taiwan, surgical intervention is the last choice for treatment. In our analysis, only 190 (52.05%) of the 365 patients underwent CCY within 60 days after previous PTGBD during index admission for acute cholecystitis. In addition, 25 patients eventually accepted CCY during follow-up, and 15 (60%) of these patients underwent laparoscopic CCY. The overall mortality rate was 19.47% in the CCY within 2 months group and 50.29% in the no CCY within 2 months group, in which the patients had higher CCI scores and older age. In terms of RBE-related mortality, five patients died in the no CCY within 2 months group, while no patients died in the CCY within 2 months group. Therefore, the CCY within 2 months group had a better survival probability in our analysis. All the details were shown in Table 2.

**Table 2:** Outcomes comparisons between patients of acute cholecystitis underwent CCY within 2 months/ No CCY within 2 months after gallbladder drainage.

**Study group**	**CCY within 2 months** ***N*** **=** **190**		**No CCY within 2 months** ***N*** **=** **175**		***P*-value**
	***N***	***SD*; %**	***N***	***SD*; %**	
**Age (*****SD*****)**	68.42	13.17	72.67	13.75	0.568
**Surgical method**					
Open CCY	102	53.68	10	5.71	N.A.
Laparoscopic CCY	88	46.32	15	8.57	N.A.
**Time to events (months)**	9.60	15.18	11.21	13.92	0.246
**RBE**					
Patients	59	31.05	81	46.29	0.003
Events	111	N.A.	173	N.A.	
Overall mortality	37	19.47	88	50.29	<0.001
RBE related mortality	0	0.00	5	2.86	0.025
**Total RBE medical expenses (NT $)**	68,561	85,343	120,284	151,225	<0.001

*SD, standard deviation; CCY, cholecystectomy; RBE, recurrent biliary event; NT $, New Taiwan dollars; N.A., not applicable*.

### RBEs

The definition of RBE included ER visits and admissions due to cholelithiasis, cholecystitis, cholangitis, and biliary pancreatitis. Although the time to the event was shorter in the subsequent CCY within 2 months group (9.6 ± 15.18 months) than in the no CCY within 2 months group (11.21 ± 13.92 months), the total number of RBEs was higher in the no CCY within 2 months group. The total number of RBEs was 111 events in 59 patients in the CCY within 2 months group and 173 RBEs in 81 patients in the no CCY within 2 months group, which showed more RBEs in the no CCY within 2 months group. This situation resulted in the total medical expenses for RBEs being much higher in the no CCY within 2 months group (68,561 ± 85,343 NT$ vs. 120,284 ± 151,225 NT$). The comparisons of medical expenses were demonstrated in Table 3.

**Table 3:** The comparisons of medical expenses between patients underwent PTGBD accept CCY within/after 2 months or without CCY.

**Variable**	**Index admission**		**CCY**		**Recurrent biliary events**		**Total charges**	
	**AVG expenses (NT $)**	***p*-value**	**AVG expenses (NT $)**	***p*-value**	**AVG expenses** ** (NT $)**	***p*-value**	**AVG expenses** ** (NT $)**	***p*-value**
CCY within 2 months (*N* = 190)	89,951 ± 103,313	<0.01	94,274 ± 91,883	0.73	68,561 ± 85,343	<0.01	172,370 ± 172,253	<0.01
CCY after 2 months (*N* = 25)	53,866 ± 40,068	<0.01	87,468 ± 91,497	Ref	119,219 ± 87,647	<0.01	190,970 ± 112,916	<0.01
No CCY (*N* = 150)	188,212 ± 329,693	Ref	0.00 ± 0.00	N.A.	120,707 ± 170,679	Ref	243,114 ± 362,431	Ref

*PTGBD, percutaneous transhepatic gallbladder drainage; CCY, cholecystectomy; NT $, New Taiwan dollars; AVG, average; ref, reference; N.A., not applicable*.

### RBE-Free Survival

**Figure 2:**
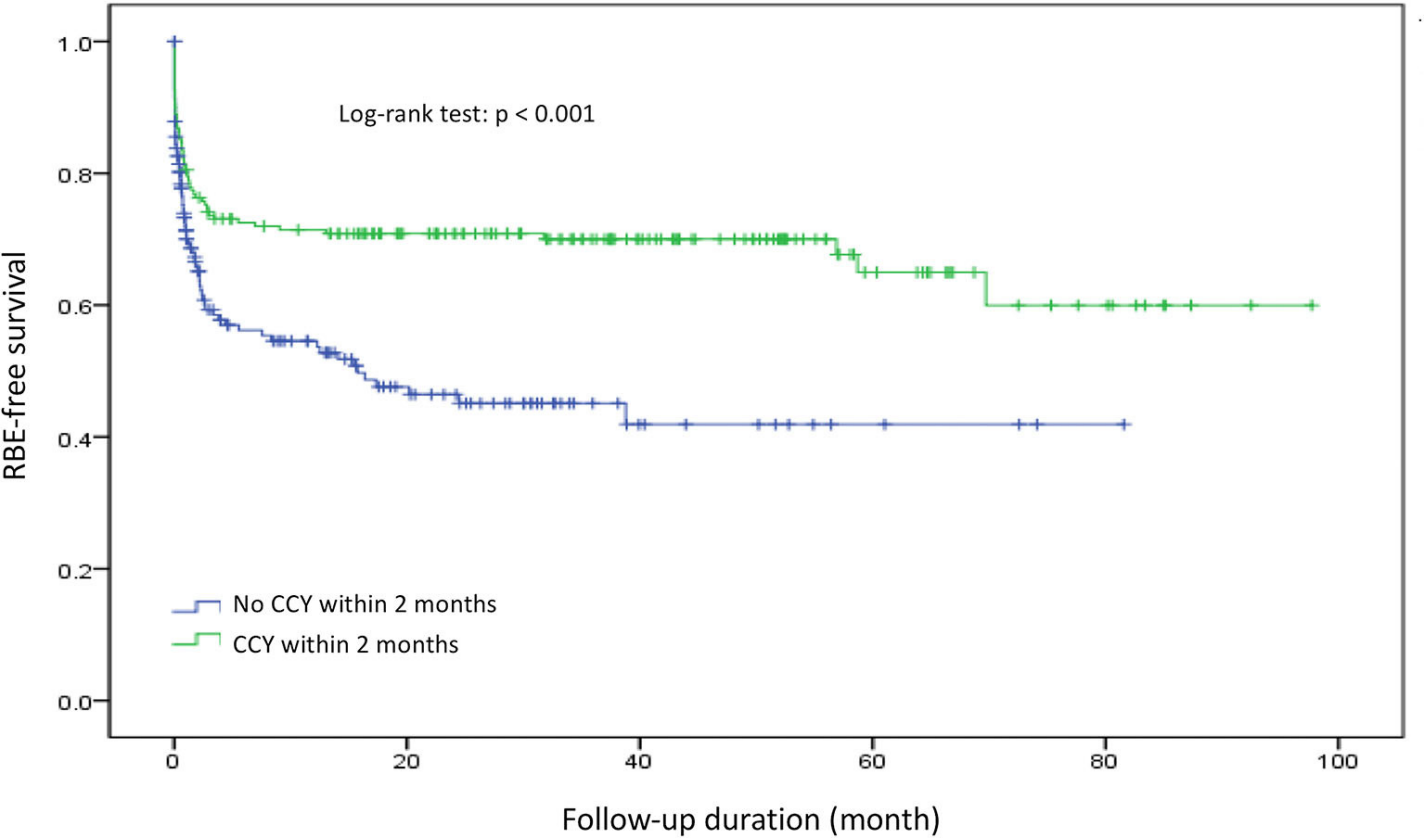
Recurrent biliary event survival comparison between the CCY within 2 months group and the no CCY within 2 months group. RBE, recurrent biliary event.

**Figure 3:**
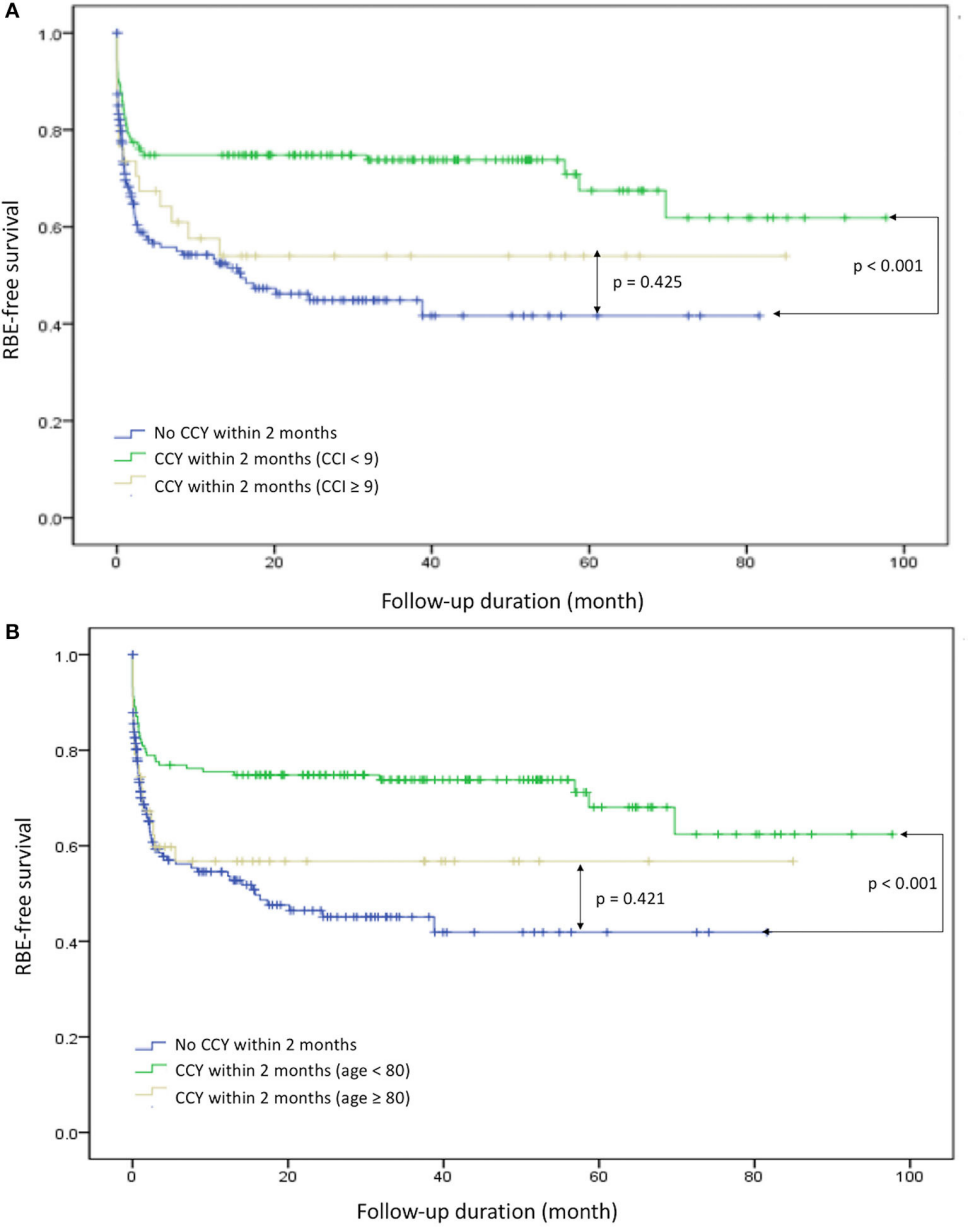
Recurrent biliary event survival comparison in specific groups. **(A)** CCI ≥ 9 and CCY < 9 with CCY within 2 months and no CCY within 2 months. **(B)** Age ≥ 80 and age < 80 with CCY within 2 months and no CCY within 2 months. RBE, recurrent biliary event; CCI, Charlson Comorbidity Index; CCY, cholecystectomy.

To evaluate the safety and protective effects of CCY after PTGBD for acute cholecystitis, we examined the RBE-free survival, which referred to both RBEs or mortality events as end points for measurements. RBE-free survival was significantly better in the CCY within 2 months group than in the no CCY within 2 months group (60 vs. 42%, *p* < 0.001). The results are shown in Figure 2. After we stratified the CCY within 2 months group patients by age and CCI score, the RBE-free survival became similar to that of the no CCY within 2 months group patients when patients were older than 80 years old (56 vs. 42%, *p* = 0.421) or had a CCI score ≥ 9 (54 vs. 42%, *p* = 0.425). Detailed information is provided in Figure 3.

### Medical Expenses

Medical expenses for the index admission, CCY, and subsequent admissions and ER visits due to RBEs and the total medical charges were calculated. We isolated the 25 patients who eventually accepted CCY at least 2 months apart from the index admission from patients without CCY within 2 months.

The average medical expenses for the index admission were 89,951 NT$, 53,866 NT$, and 188,212 NT$ in the CCY within 2 months group, CCY after 2 months group, and no CCY group, respectively. The much higher medical expenses in the no CCY group indicated the complicated clinical condition and significant comorbidities in this group of patients. For the medical expenses of RBEs, the average expenses were 120,707 NT$ in the no CCY group, which was much higher than the 68,561 NT$ in the CCY within 2 months group.

The average total medical costs were 243,114 NT$, 190,970 NT$, and 172,370 NT$ in the no CCY group, CCY after 2 months group, and CCY within 2 months group (*p* < 0.01), which suggests the later CCY was performed, the higher the medical expenses were. The medical cost comparisons are shown in Table 3.

## Discussion

Reflecting the culture in Taiwan, surgical intervention was always the last choice for treatment. In our analysis, only 190 (52.05%) of the 365 patients underwent CCY within 60 days after previous PTGBD during the index admission for acute cholecystitis. Although previous studies showed that CCI score and the American Society of Anesthesiologists physical status classification (ASA-PS) can be used for surgical risk classification (34) in acute cholecystitis evaluation (35, 36), some reports showed similar survival benefits and lower complication rates using laparoscopic CCY compared to initial PTGBD in high risk acute cholecystitis patients (37). No adequate risk evaluation has ever been explored in second stage CCY after PTGBD.

In our study, the data were collected for patients who underwent PTGBD for high risk acute cholecystitis (from a retrospective database that included a random selection of ~5% of the Taiwan population), and only 4.08% of patients underwent PTGBD instead of CCY for acute cholecystitis treatment at the index admission. This observation indicates that CCY is the favored treatment method for acute cholecystitis in Taiwan and compatible with the guideline suggestion worldwide.

According to other investigations into RBEs after patients accepted PTGBD without further CCY, the cumulative incidence of biliary events was ~30% (38). In our analysis, the proportion of patients who experienced RBEs was 31.05% in the CCY within 2 months group and 46.29% in the no CCY within 2 months group. Our RBE rate is higher than that of other analyses because not all of our patients accepted CCY within 2 months as a series treatment algorithm; some of them underwent CCY due to RBEs in a short time period after the index admission. Another reason is the follow-up duration was significantly longer in the CCY within 2 months group than in the no CCY within 2 months group (39.68 vs. 20.26 months, *p* = 0.047), which led to higher RBEs in comparison. Even in this setting, the RBEs, RBE-related medical expenses, and RBE-related mortality rate were significantly lower in the CCY within 2 months group. This finding confirmed that CCY can reduce RBEs after PTGBD treatment in high risk acute cholecystitis patients.

### RBE-Free Survival

Due to improvements in technology, equipment, and surgical skills, CCY, either early CCY or delayed CCY, are the standard treatment of choice in acute cholecystitis. We focused on which patients may not benefit from the current aggressive treatment methods for acute cholecystitis. A large scaled study have been shown elderly is not a problem for CCY (39), but the definition of elderly is too young for modern society in our opinion. As we further evaluated the RBE-free survival in the CCY within 2 months group using age≥65 vs. age < 65, age ≥ 75 vs. age < 75, age ≥ 80 vs. age < 80, CCI score ≥ 6 vs. CCI score < 6, CCI score ≥ 7 vs. CCI score < 7, CCI score ≥ 8 vs. CCI score < 8, and CCI score ≥ 9 vs. CCI score < 9, we found that when patients were older than 80 years or their CCI score exceeded nine, the benefits of RBE-free survival became insignificant in the CCY within 2 months group.

### Medical Expenses

The financial impact was considered, and the patients who initially underwent PTGBD without further CCY had the highest medical costs, while the patients who initially underwent PTGBD and accepted CCY within or after 2 months from the index admission had similar total medical costs. Whether patients eventually underwent CCY or not, the medical expenses due to RBEs were higher in the no CCY within 2 months group than in the CCY within 2 months group. Earlier CCY after PTGBD decreased medical expenses.

There are limitations to our study. For example, comorbidities associated with retrospective database analysis-related selection bias may confound the assignment to CCY. This selection resulted in patients of younger age with less comorbidities and conceivably better performance status in the CCY within 2 months group than in the no CCY within 2 months group. This bias makes our results more reliable if the subgroup analysis showed no benefits over patients in the CCY within 2 months group than in the no CCY within 2 months group, because patients with better conditions in the same CCI score are suggested to accept CCY clinically. Another limitation is that we could not determine if the patients accepted CCY within 2 months as a series treatment algorithm after PTGBD or just accepted CCY at another cholecystitis event within 2 months because of the limitations of the NHIRD. Because whether high risk acute cholecystitis patients who undergo PTGBD accept series CCY depends on personal choices, further prospective randomized studies are not a possible solution because this limitation would be present in similar studies. Future prospective multi-center studies can clarify and avoid the selection bias in different study groups.

## Conclusions

We confirmed CCY after PTGBD can reduce RBEs, RBE-related medical expenses, and the RBE-related mortality rate in high surgical risk patients with acute cholecystitis. In patients who underwent PTGBD, the RBE and survival benefits of subsequent CCY within 2 months became insignificant in patients older or equal than 80 years old or with a CCI score ≥ 9.

## Data Availability Statement

The raw data supporting the conclusions of this article will be made available by the authors, without undue reservation.

## Ethics Statement

The studies involving human participants were reviewed and approved by IRB of Chung Shan Medical University Hospital. Written informed consent for participation was not required for this study in accordance with the national legislation and the institutional requirements.

## Author Contributions

C-CL and M-CT: designed and carry out this study. C-CW, C-CL, and M-CT: conception and design. M-HT, S-WW, and T-WY: acquisition of data. C-CW, S-WW, and W-WS: analysis and interpretation of data. C-CW and Y-TW: drafting of the manuscript. H-LL and B-HS: critical revision of the manuscript. W-WS and M-HT: statistical analysis. M-HT, C-CL, and M-CT: supervision. All authors reviewed the manuscript.

## Conflict of Interest

The authors declare that the research was conducted in the absence of any commercial or financial relationships that could be construed as a potential conflict of interest.
